# Predicting Quality of Life of Patients in Romania with Heart Failure with Preserved Ejection Fraction by Analyzing H2FPEF Scores

**DOI:** 10.3390/healthcare13080909

**Published:** 2025-04-15

**Authors:** Alina Ciobanu-Teașă, Suzana Guberna, Cosmina Elena Jercălău, Octavian Ceban, Cătălina Liliana Andrei, Crina Julieta Sinescu

**Affiliations:** 1 Department of Cardiology, University of Medicine and Pharmacy “Carol Davila”, Emergency Hospital “Bagdasar-Arseni”, 020021 Bucharest, Romania; cosmina-elena.jercalau@drd.umfcd.ro (C.E.J.); catalina.andrei@umfcd.ro (C.L.A.); crina.sinescu@umfcd.ro (C.J.S.); 2 Economic Cybernetics and Informatics Department, The Bucharest University of Economic Studies, 010552 Bucharest, Romania; octavianceban1995@gmail.com

**Keywords:** quality of life, heart failure, chronic disease management, risk stratification, H2FPEF, patient-centered care

## Abstract

**Background/Objectives**: This study aimed to find a way to predict the quality-of-life factors for patients with heart failure with preserved ejection fraction based on their H2FPEF scores. **Methods**: We performed a prospective observational analysis of 142 hospitalized patients diagnosed with HFPEF who were followed for 12 months after discharge. We calculated the H2FPEF score for each patient during hospitalization. The follow-up after discharge aimed to monitor limitations of usual physical activity, recently experienced fatigue, the presence of leg edemas, the ability to exercise regularly, and sadness. We thus obtained data about these patients’ quality of life, their physical and mental limitations, their number of readmissions, and the percentage of mortality. We used logistic regression models to estimate the relationship between the H2FPEF score and each variable, providing probabilities for each sign or symptom of the disease mentioned by the patients. **Results**: All the observed variables showed statistical significance. Marked limitations of physical activity showed the strongest relationship with the H2FPEF score, followed by edema and regular exercise. **Conclusions:** Our research shows a method with which to predict the quality-of-life (QoL) factors in patients with HFPEF based on their H2FPEF scores. We can predict which patients are at high risk and require more medical resources by quickly calculating their H2FPEF scores.

## 1. Introduction

Heart failure with preserved ejection fraction (HFPEF), also known as diastolic heart failure, accounts for up to 54% of heart failure (HF) cases. HFPEF results from high left-ventricular (LV) filling pressure despite a normal LV ejection fraction (LVEF of ≥50 percent) [1,2,3,4]. A patient diagnosed with this disease has a 20% chance of surviving another 10 years. The prognosis is worse than for most forms of cancer [4,5]. The burden of the disease is felt by patients, in their decreased quality of life and functional disability; by their families, due to the need to take care of them and the patient being dependent on a family member for regular physical activity; and by society, as patients are often unable to work and are socially marginalized due to the lack of activities adapted to the disease [6,7,8,9].

Cardiac recovery and rehabilitation programs are beginning to be developed in Romania, but also globally. These programs are needed because patients hospitalized for heart failure often return home upon discharge to the same environmental conditions, with insufficient medical education on the correction of the precipitating risk factors. Moreover, returning home makes it harder to follow these patients, at least in the short term, when they are in the vulnerable period of the disease [9,10,11]. Currently, the data on the at-home quality of life of these patients are limited [12,13,14]. Telemedicine is a quick solution until the complete development of such programs and centers. In the management of the disease, it is useful to follow patients at home to enable quick intervention with therapeutic measures before HF decompensation.

In our study, we identified the most vulnerable patients with the highest risk of HF decompensation during hospitalization. After discharge, we tracked the most common signs and symptoms of the disease that can be easily identified by the patient and reflect their quality of life, such as difficulty performing regular physical activities, the presence of a leg edema, or the inability to exercise, via a telephonic questionnaire. In the final step, the H2FPEF score calculated for each patient at the time of admission was correlated with the signs and symptoms mentioned.

The H2FPEF score is a tool to estimate the probability of HFPEF. This score stratifies the likelihood of HFPEF into low, moderate, and high categories based on the clinical characteristics and echocardiographic findings. Current studies on the quality of life of patients with HF have used questionnaires, such as The Minnesota Living with Heart Failure Questionnaire (MLHFQ) and The Kansas City Cardiomyopathy Questionnaire (KCCQ) [15,16,17,18].

Our method was to ask about the factors that patients could easily appreciate and which concerned their quality of life, emphasizing the signs and symptoms of heart failure decompensation.

Our objective was to validate whether the hypothesis that the H2FPEF score can predict the quality-of-life factors is true and if so, to use this information to take the necessary steps to improve patients’ quality of life. Being able to anticipate the evolution of quality of life enabled us to take proactive measures in patient treatment.

There are limited studies on the H2FPEF score, and they provide data on the prognosis of the disease and the association between ischemic heart disease and comorbidities [11,19,20,21]. We wanted to predict which patients would be more vulnerable at home and which would require more frequent medical check-ups from the moment they entered the hospital. We obtained statistically significant predictions of the patients’ quality of life at home using their H2FPEF scores. Moreover, we show that follow-up can be performed via telemedicine, which is an accessible, cheap, and effective solution that can help enhance patients’ experiences and health conditions.

## 2. Materials and Methods

### 2.1. Study Design and Study Population

This prospective, observational study was conducted in the Cardiology Department of the “Bagdasar-Arseni” Emergency Hospital in Bucharest, Romania, during an enrollment period of 1 year and 6 months, from May 2021 to October 2022, and followed the patients’ evolution after discharge for 12 months, from May 2022 to April 2023. This study was conducted on a sample of 142 patients over 18 years old and diagnosed with preserved LVEF. The cohort comprised patients admitted with a diagnosis of heart failure with preserved ejection fraction (LVEF ≥ 50 percent).

In Romania, the public health system includes emergency hospitals, as well as hospitals for chronic diseases. In each of these, patients can be admitted for evaluation and treatment. The outpatient system does not have the capabilities to provide a complex evaluation (echocardiography, NT pro BNP dosage, lung X-Ray, other blood tests, etc.) for all patients in each of these hospitals. Thus, patients with compensated heart failure are scheduled for hospitalization to evaluate and monitor the evolution of the disease under treatment [22,23,24,25].

The follow-up was carried out via a phone call during the first year after discharge due to the difficulty of follow-up in the cardiology clinic in the context of the recent SARS-CoV-2 virus pandemic.

The inclusion criteria were as follows: (1) patients over 18 years old; (2) patients with signs/symptoms of heart failure; (3) patients with NT-proBNP ≥300 pg/mL; (4) patients with LVEF (left-ventricular ejection fraction) ≥50% objectified echocardiographically; (5) patients with only signs and symptoms specific to the disease; (6) patients with compensated or acute heart failure; and (7) patients who signed the informed consent form and agreed to the prospective follow-up and to participate in the evaluation via telephone.

The exclusion criteria were as follows: (1) patients with severe valvular diseases; (2) patients with severe neuropsychiatric diseases; (3) patients with severe liver, kidney, or lung diseases; (4) patients with ongoing infections; (5) patients diagnosed with autoimmune diseases or malignancy; (6) patients with moderate and severe anemia; and (7) patients with hope of survival of less than 1 year (as this study aimed to observe the evolution for 12 months after discharge, which may not have been feasible for these patients).

All the patients had venous blood samples collected within 30 min of admission. The NT-proBNP level was evaluated using the ELISA method with a PATHFAST compact autoanalyzer. The LVEF (left-ventricular ejection fraction) values were obtained using transthoracic echocardiography performed during hospitalization. The modified Simpson biplane method was used to calculate the left-ventricular end-systolic volumes (LVESVs) and end-diastolic volumes (LVEDVs) from 4- and 2-chamber views. The LV volumes were corrected for body surface area.

The demographic data included age, gender, emergency admission or appointment, NYHA class, normal or arterial hypertension, atrial fibrillation, and overweight and obesity. The mean, minimum, and maximum ages of the patients enrolled in this study were 73.1, 40, and 93, respectively (Table 1).

**Figure 1:**
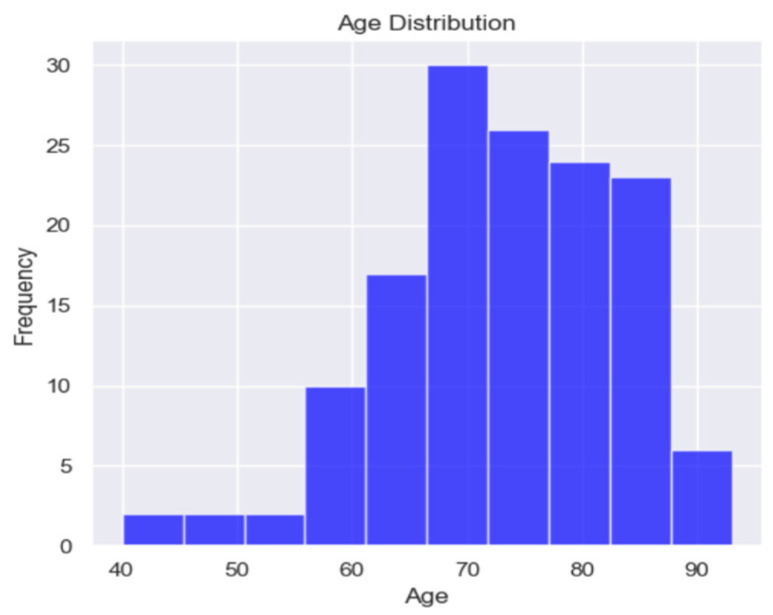
Age distribution of studied population.

In this study, only 13 patients were under 60 years old (Figure 1), suggesting a prevalence of the disease in elderly patients, a characteristic of HFPEF.

The NYHA class distribution at presentation was 58% for NYHA class III, and 42% for NYHA class IV (Table 2). Patients in NYHA class III/IV had typical signs and symptoms of acute decompensated HF.

**Figure 2:**
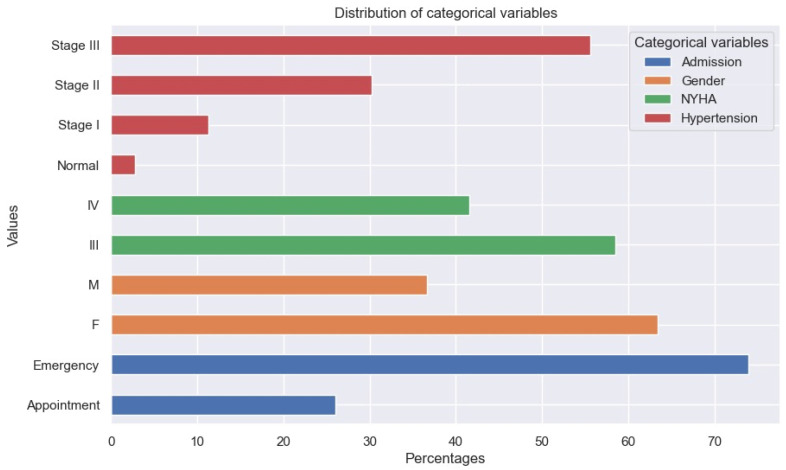
Distribution of categorical variables.

The percentage of women with HFPEF was higher in the group of patients studied, as it is known that HFPEF is more frequent in women (Figure 2).

**Figure 3:**
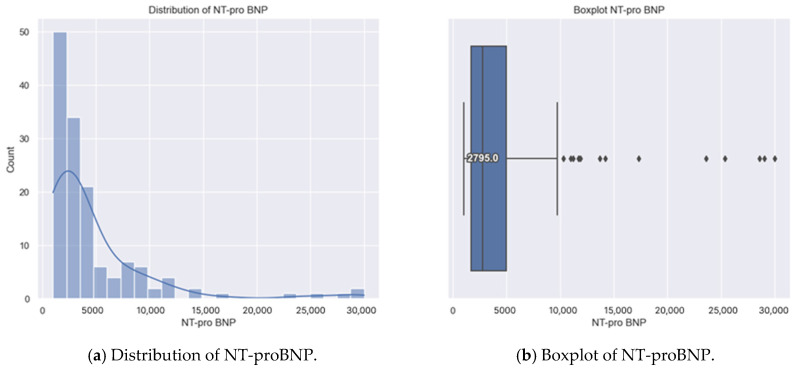
(**a**,**b**) Distribution of NT-proBNP.

The distribution of NT-proBNP levels revealed that most patients had relatively low values, ranging from 1.005 pg/mL to 5008 pg/mL, encompassing 75% of the sample (Table 3). The remaining 25% (top quartile) had levels exceeding 4.716 pg/mL. The data also included outliers, with values exceeding 10.000 pg/mL and a maximum recorded value of 30.000 pg/mL (Figure 3).

The tracked quality-of-life factors were as follows: a marked limitation of physical activity, edema, recent fatigue, the ability to exercise, palpitations, and sadness.

All the variables used were binary, as described in the following summary statistics table (Table 4).

These variables were chosen to describe patients’ quality of life because they directly impact lifestyle and show the symptoms and limitations caused by heart failure decompensation. For example, the presence of pitting edema, marked limitation of physical activity, and fatigue at regular exertion indicate an exacerbation of the disease. These patients should be medically reassessed as soon as possible.

The inability to exercise daily is a parameter associated with the limitations of the disease and contributes to its unfavorable evolution.

Illness indicates physical and mental limitations, so we wanted to identify which patients were sad and could benefit from psycho-emotional support.

We considered it useful to use some parameters from the patients’ hospitalizations to determine if there was a relationship between them and their H2FPEF scores. One variable was the presence of palpitations. This parameter is a symptom of the disease and simultaneously contributes to the course of the disease. It suggests a possible cause—atrial fibrillation—which must be investigated and treated if present.

The distribution of the independent variable used in this research, the H2FPEF score, a specific indicator of heart failure with preserved ejection fraction, is presented below.

**Figure 4:**
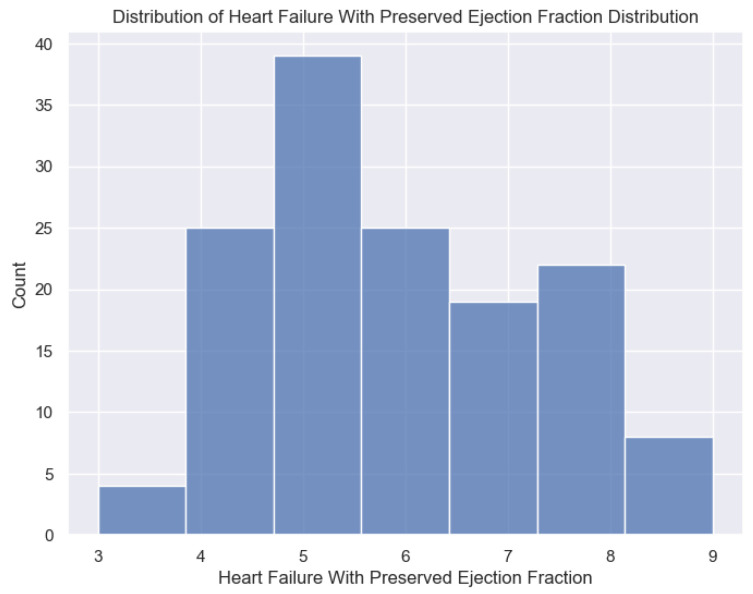
Distribution of H2FPEF scores.

The distribution of the H2FPEF scores in the sample is illustrated in Figure 4. The data reveal a concentration of scores between 4 and 6, characteristic of this patient population, with relatively few individuals scoring at the extremes of 3 or 9.

Patients admitted with HFPEF had a minimum hospital stay of 3 days, a maximum of 9 days, and an average of 5.9 days (Table 5).

The patients received the following treatments during hospitalization and after discharge: intravenous loop diuretics and then oral loop diuretics, ACEIs (Angiotensin-converting enzyme inhibitors)/ARBs (Angiotensin receptor blockers), spironolactone, beta-blockers, and digoxin (Table 6). In total, 24% of patients required intravenous loop diuretics.

### 2.2. Study Method

Quality-of-life data were obtained one year after discharge via a telephone interview in which patients answered the questions below. The average duration of the phone call was 15 min. We aimed to obtain data on the following variables that describe the quality of life: marked limitation of physical activity, pitting edema, recent fatigue, ability to exercise, palpitations, and sadness.

For the variables obtained from the answers provided during the phone interview, we recorded and analyzed data for 126 patients. The other 16 patients died within the first year after discharge.

The questions for the phone interview were as follows:

1. In the last month, have you breathed harder during physical efforts?
2. Do you have pitting edemas?
3. Are you feeling more tired lately?
4. Do you manage to exercise minimum 30 min/day, minimum 5 days/week?
5. Are you sad? Is your sadness caused by the cardiovascular disease?
6. Do you have palpitations?
7. Have you been admitted to a cardiology ward in the last year? If the answer was Yes, the reason for hospitalization was also specified.

Only the answers that were related to the evolution of heart failure were considered.

The questions were designed in such a way that the answers could be converted to Yes or No values, to ensure a fast data collection process and to be able to apply the linear regression statistical method, a suitable tool for the analysis of variables with binary values. The existing questionnaires (MLHFQ and KCCQ) that measure quality-of-life allow for multiple answers and would not have allowed for the type of analysis we intended to perform.

We tried to find a correlation between the answers provided and the H2FPEF score value from admission to be able to predict which patients were at higher risk and, thus, identify the vulnerable ones early. These patients require closer follow-up via frequent check-ups with a general practitioner or cardiologist. They and their families should be medically educated about the precipitating factors and the signs and symptoms of the disease’s decompensation to reach their doctor faster.

The questionnaire was developed and used exclusively for this study. It includes questions whose answers helped us test our working hypothesis that H2FPEF score can provide a prediction of quality-of-life factors. Developing a new standard questionnaire to assess quality of life was not within the scope of this study.

This study was conducted according to the guidelines of the Declaration of Helsinki and approved by the Ethics Committee of “Carol Davila” University of Medicine and Pharmacy, Bucharest, Romania (protocol code: PO-35-F-03; 1 October 2021).

### 2.3. Statistical Analysis

Logistic regression analysis was conducted to better understand the impact of heart failure with preserved ejection fraction score (H2FPEF) on binary quality-of-life indicators.

Logistic regression is defined as a supervised machine learning algorithm that accomplishes binary classification tasks by predicting the probability of an outcome, event, or observation. The logistic regression method was selected due to its robustness for this type of sample and its strong interpretability. This approach allowed for the estimation of each indicator’s probability based on the H2FPEF score, assessing the statistical significance via the associated *p*-values. A *p*-value threshold of 0.05 was used. The model was considered statistically significant if the *p*-value was below the threshold. The research design involved running separate logistic regressions for each life-quality indicator, treating the H2FPEF score as the independent variable and the indicators as the dependent variables. All analyses were performed using Python (version 3.11), utilizing standard statistical packages, such as Pandas, Statsmodels, and Scikit-learn.

## 3. Results

This section presents the results of the logistic regression analyses, highlighting the impact of the H2FPEF score on the probability of the studied variables.

### 3.1. Marked Limitation of Physical Activity

The H2FPEF score had a pronounced effect on the likelihood of experiencing limitations of physical activity. The coefficient was 0.5708, indicating a positive relationship, with a *p*-value that was very close to 0, suggesting strong statistical significance (Table 7).

**Figure 5:**
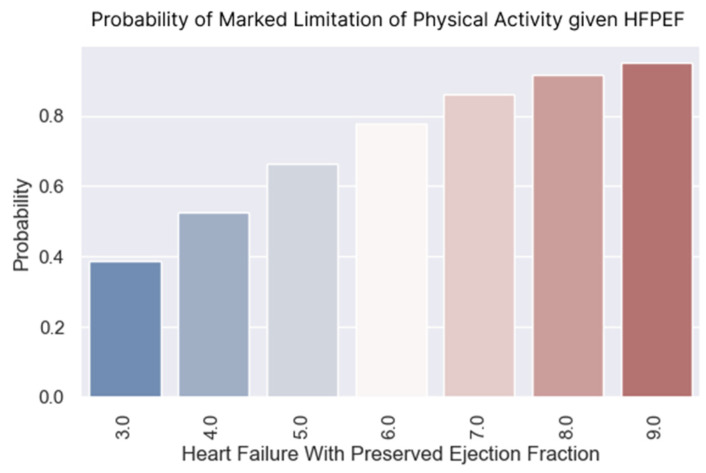
The probability of having a limitation of physical activity associated with each score.

The higher the H2FPEF score, the higher the probability of not being able to perform usual physical activities. Thus, with an H2FPEF score of only four points, approximately 50% of the patients could not perform their usual physical exertion. With a score of seven points, over 80% of the patients experienced significant limitations to their physical activity (Figure 5).

**Figure 6:**
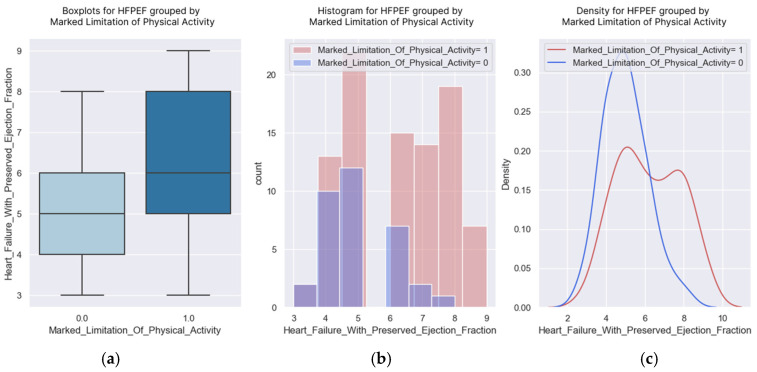
(**a**–**c**) H2FPEF scores grouped by marked limitation of physical activity.

The limitation of physical activity caused by the disease is highlighted in the diagrams above (Figure 6). A higher score was correlated with a greater probability of the patients experiencing important limitations.

### 3.2. Edema

**Figure 7:**
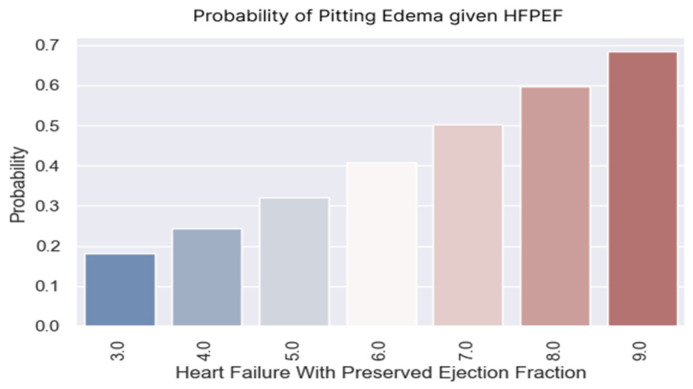
Probability of having edema based on H2FPEF score value.

An edema is a sign of heart failure that can be easily identified by patients. Weight gain and signs, such as a pitting edema, should alert a patient to go to the doctor as soon as possible for an adjustment of their therapeutic regimen. Knowing which patients could develop a pitting edema, based on to their H2FPEF scores (Table 8, Figure 7), would be useful for preventing the decompensation of the disease.

**Figure 8:**
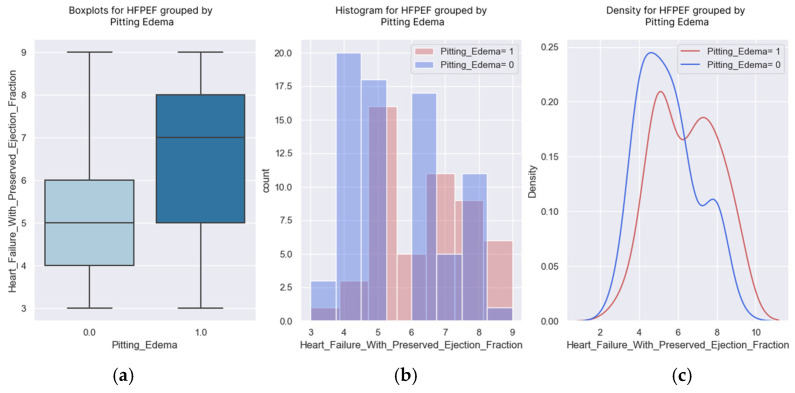
(**a**–**c**) Boxplots and diagrams for pitting edema and H2FPEF scores.

The probability of having an edema increased as the score increased (Figure 8). The patient with an H2FPEF score of seven points had a probability of 50% of having a pitting edema.

### 3.3. Recent Fatigue

**Figure 9:**
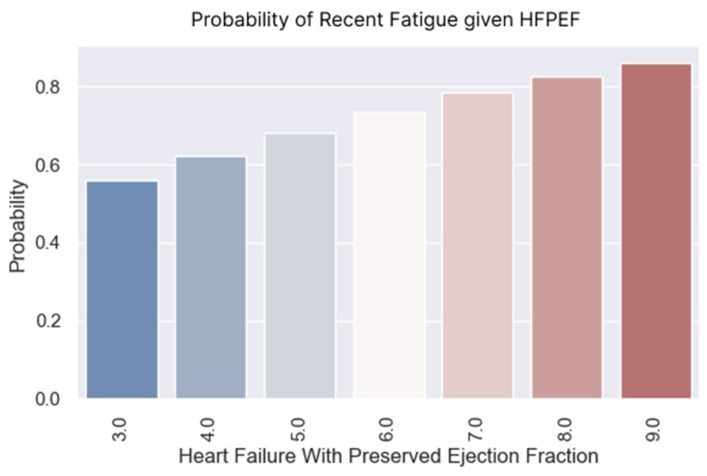
Probability of experiencing fatigue based on H2FPEF scores.

**Figure 10:**
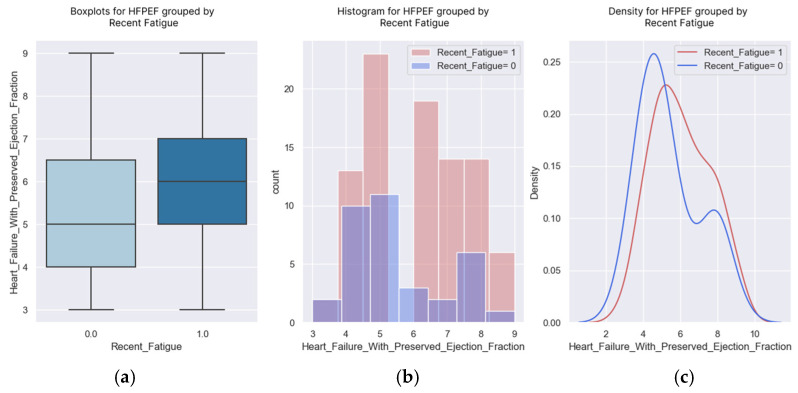
(**a**–**c**) Boxplots and diagrams for fatigue and H2FPEF score.

Fatigue is another indicator of the disease that we tracked during the phone interview. The experience of fatigue and its presence during small efforts indicate the decompensation of the disease. The logistic regression demonstrated that the probability of experiencing fatigue rose as the H2FPEF score increased (Figure 9 and Figure 10). The regression coefficient was positive (0.2615), with a corresponding *p*-value of 0.051 (Table 9), indicating statistical significance.

Almost all the patients with a score of nine points felt tired at home. Their fatigue was caused by the disease. The probability of fatigue at regular exertion was greater than 50% for the patients with an H2FPEF score of four points.

### 3.4. Regular Exercise

The indication to exercise regularly, within the limit of personal tolerance and with a gradual increase in effort, is received by all patients with heart failure. However, the disease, readmissions, diuretic medication, and physical deconditioning limit patients’ abilities to exercise regularly.

The sustained medical education of patients and their family members and participation in cardiac recovery programs can improve patients’ physical capacities.

The logistic regression demonstrated that the probability of exercising decreased as the H2FPEF score increased. The regression coefficient was negative (−0.3371), with a corresponding *p*-value of 0.02, indicating statistical significance (Table 10).

Only 17% of the patients with a score of seven points exercised regularly.

**Figure 11:**
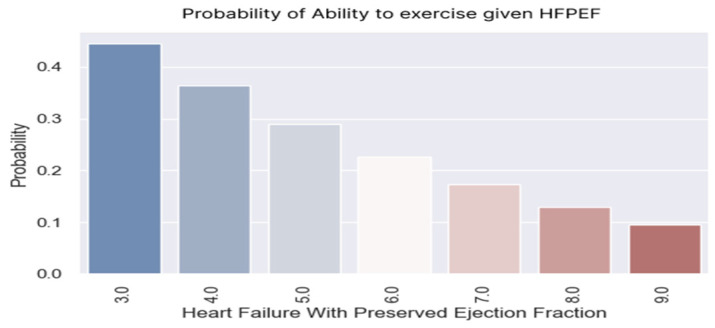
Probability of exercising regularly according to H2FPEF score values.

**Figure 12:**
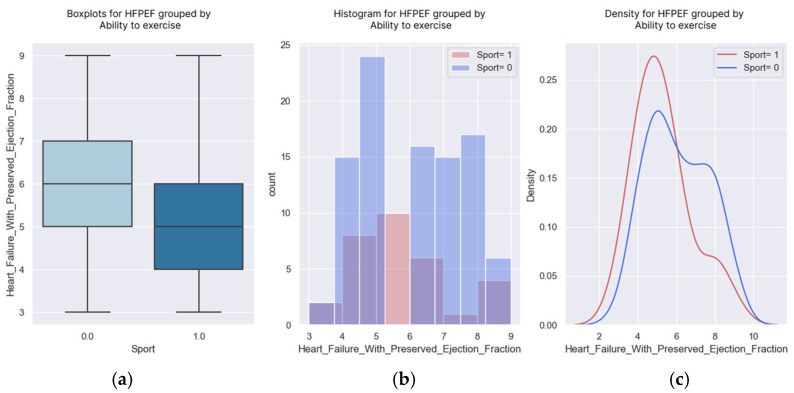
(**a**–**c**) Boxplots and diagrams for regular exercise and H2FPEF scores.

One in two patients with an H2FPEF score of three points managed to perform the recommended regular exercises. A patient with a score of nine points had a probability of exercising lower than 10% (Figure 11 and Figure 12).

Knowing these probabilities, we must instruct these patients to participate in cardiac recovery programs to improve their physical capacities.

### 3.5. Sadness

HFPEF is a disease with many physical, social, and mental limitations, so patients with heart failure are prone to being sad. The improvement of a patient’s mental state has beneficial effects on the evolution of disease. The patient will show up on time for medical check-ups, administer their treatment correctly and completely, and perform the recommended regular physical activity.

The regression coefficient was positive (0.3481), with a corresponding *p*-value of 0.009, indicating statistical significance (Table 11).

**Figure 13:**
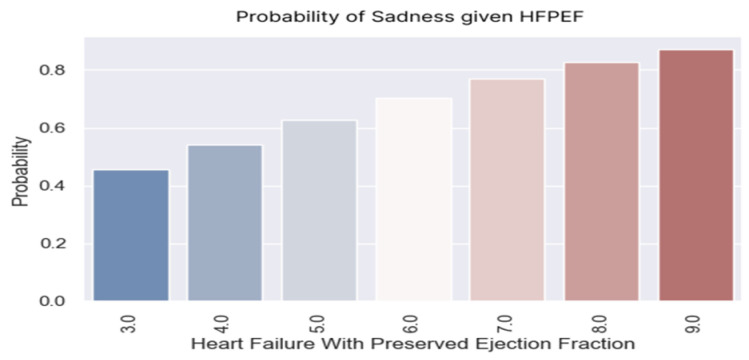
Probability of sadness according to H2FPEF scores.

**Figure 14:**
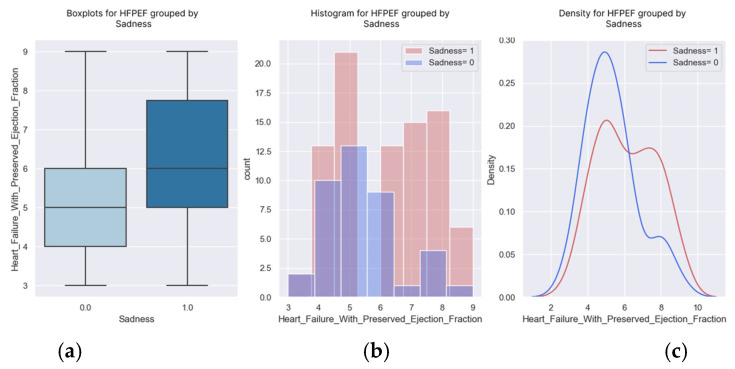
(**a**–**c**) Boxplots and diagrams for sadness and H2FPEF scores.

The likelihood of feeling sad increased as the score increased. The patients with an H2FPEF score of seven points had a more than 70% probability of being sad. According to Table 11 and the graphs (Figure 13 and Figure 14), the H2FPEF score can be a good predictor of sadness. Psycho-emotional counseling measures can be initiated by knowing which patients are vulnerable.

### 3.6. Palpitations

The logistic regression below demonstrates that the probability of experiencing palpitations rose as the H2FPEF score increased. The regression coefficient was positive (0.3374), with a corresponding *p*-value of 0.003 (Table 12), indicating statistical significance.

**Figure 15:**
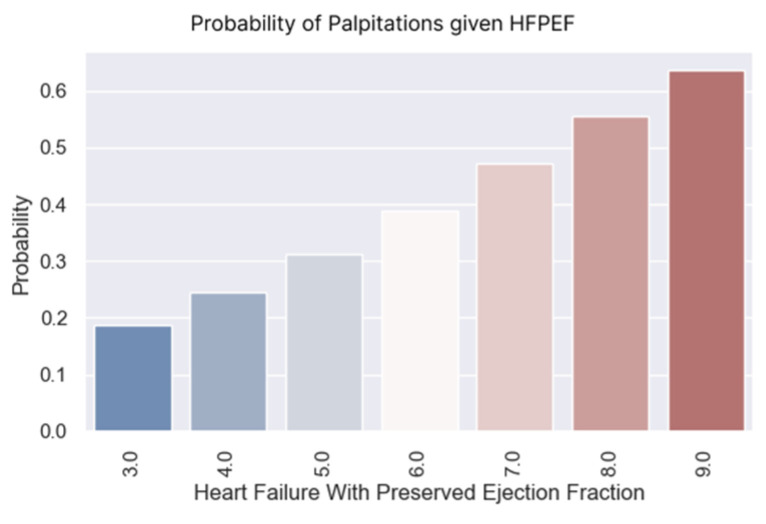
Probability of palpitations based on H2FPEF scores.

The following probabilities were derived from the estimated model to gain a clearer understanding of the probability associated with each score value (Figure 15).

It is important to know which patients have palpitations. They are caused by the decompensation of the disease and the possible onset of atrial fibrillation. Detecting palpitations early is beneficial because measures to control the rhythm and heart rate and prevent cardioembolic complications can be taken quicker.

**Figure 16:**
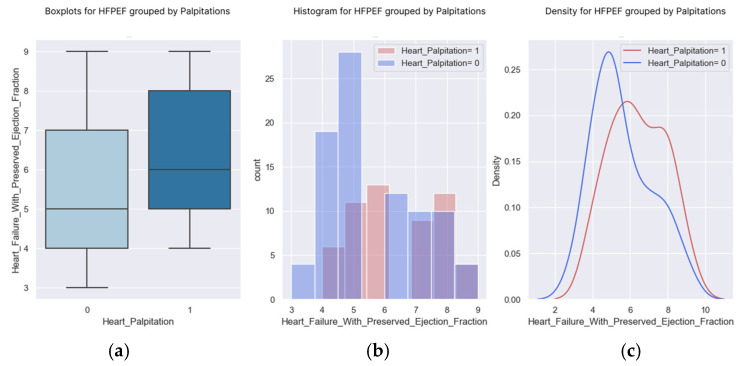
(**a**–**c**) Boxplots and diagrams for palpitations and H2FPEF scores.

According to the obtained results (Table 12 and Figure 15 and Figure 16), we expect a patient with a high H2FPEF score to have palpitations and require additional treatment to improve their prognosis.

### 3.7. Comparing Results

The table below (Table 13) displays the coefficients from the logistic regression along with their associated *p*-values, providing a comprehensive summary of the relationships outlined previously. All the variables exhibited statistical significance. The strongest relationship with the H2FPEF score was observed for marked limitations of physical activity, followed by edema and sadness. We also examined the correlation between the NT-pro BNP levels and the H2FPEF score, and found that only 2.5% of the variation in the NT-pro BNP could be attributed to fluctuations in the H2FPEF score. There was a low correlation between these two variables (*p*-value = 0.06), indicating that an increase in the H2FPEF score tended to correspond with a rise in the NT-pro BNP levels.

**Figure 17:**
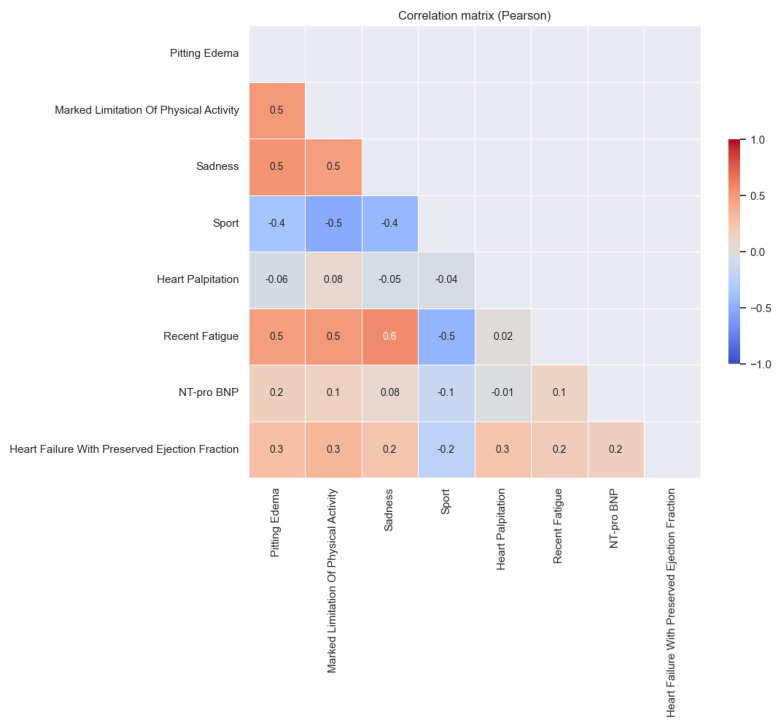
Correlation matrix of the H2FPEF score, the quality-of-life factors, and the NT-proBNP.

This correlation matrix shows a comprehensive overview of the relationship between the studied variables (Figure 17). The correlations between the quality-of-life factors and the H2FPEF score is reconfirmed. The slight association between the H2FPEF score and NT-proBNP is also been reaffirmed (0.2). The relationship between the NT-proBNP and quality-of-life factors is also examined. A low correlation is observed with edema (0.2), while no correlation is noted with palpitations (−0.01). While the NT-proBNP is recognized as a significant biomarker for HFPEF, our findings do not reveal a substantial statistical correlation between the NT-proBNP levels and quality-of-life indicators. The fact that only a slight correlation is observed between the NT-proBNP levels and the other parameters can be attributed to several factors. This study’s cohort was drawn from a single hospital unit and comprised 142 patients. There was considerable variation in their NT-proBNP levels, which ranged from 1000 pg/mL to 30,000 pg/mL. Notably, 75% of the patients had levels between 1.005 pg/mL and 5008 pg/mL. Other factors that contributed to the reduction in the NT-proBNP levels included overweight, obesity, and ongoing treatment with cardioactive drugs, such as ACE inhibitors, spironolactone, B blockers, and diuretics.

## 4. Discussion

### 4.1. Study Limitations

The limitations of our study are related to our only enrolling patients from a single medical center and for a relatively small number. This emphasizes the need for further studies to more accurately estimate the correlation between the H2FPEF score, NT- pro BNP, and quality of life. Additional questions could be added to the questionnaire for the assessment of the disease. Here, the number of questions was smaller because the interview was conducted over the phone. In addition, the long-term follow-up with these patients is needed.

Telemedicine helped us to follow these patients in the context of the difficult access to the clinic during the SARS-CoV-2 virus pandemic. However, this had limits, resulting in an incomplete medical examination.

Despite these limitations, our study highlights the importance of the H2FPEF score for risk stratification to actively intervene in vulnerable patients.

### 4.2. Future Directions

Telemedicine needs to be further developed, and this study has shown its importance. It must be accessible to both medical staff and patients. It is necessary to communicate with the patient, to find out how they feel, to teach them about their disease, to teach them how to live to prevent decompensation, to know what the signs of the evolution of the disease are, and to be able to go to the hospital quicker if necessary. Complications and an unfavorable evolution of the disease can, thus, be prevented or mitigated.

Combining machine learning technologies to understand the aggravating factors and assess patient risk with continuous monitoring via telemedicine can enhance lifespan.

## 5. Conclusions

Our study introduces a quantitative analysis of the relationship between the H2FPEF score and quality-of-life factors using statistical methods. The score offers an indication of patients’ quality of life, and our study aimed to demonstrate practically this relationship, in percentages and values, to ensure its accuracy, correctness, and statistical validation.

We obtained an important statistical correlation between the H2FPEF score and all the parameters investigated (simple indicators for a patient).

In addition, we created a profile of a high-risk patient who will require active follow-up, i.e., a patient with a high H2FPEF score who experiences a marked limitation of physical activity, edema, recent fatigue, and inability to exercise.

In daily clinical practice, we can use the results obtained by calculating each patient’s H2FPEF score and, thus, estimate how they will feel at home after discharge.

Thus, with a method for predicting the quality of life of patients at home and understanding which ones are at risk of decompensation from the disease, we can intervene early via the sustained medical education of the patients and their families through cardiac recovery programs.

We need to further develop telemedicine so that human and financial resources are used more effectively by detecting the signs and symptoms of heart failure decompensation early.

## Figures and Tables

**Table 1:** Age of studied population.

	Age
Count = 142	
Mean	73.1
Std	9.92
Min.	40
Max.	93
25%	67
50%	73
75%	81

**Table 2:** Demographic and clinical characteristics of studied population.

Variable	Prevalence %
Female	63%
Male	37%
Admission emergency	74%
Admission appointment	26%
NYHA class III	58%
NYHA class IV	42%
Normal blood pressure	3%
Hypertension stage I	11%
Hypertension stage II	30%
Hypertension stage III	56%
Atrial fibrillation	46%
Overweight and obesity	78%

**Table 3:** NT-proBNP values of studied population.

	NT-proBNP (pg/mL)
Count = 142	
Mean	4716.4
Std	5373.5
Min.	1005
Max.	30,000
25%	1712
50%	2795
75%	5008.7

**Table 4:** All variables used in this research.

Variable	Count	True	False	True Percentage
Pitting edema	126	50	76	39%
Palpitations	126	49	77	38%
Recent fatigue	126	93	33	73%
Marked limitation of physical activity	126	95	31	75%
Sadness	126	87	39	69%
Ability to exercise	126	31	95	24%

**Table 5:** Distribution of number of days of hospitalization.

	Days of Hospitalization
Count = 142	
Mean	5.9
Std	1.58
Min.	3
Max.	9
25%	5
50%	6
75%	7

**Table 6:** Percentage distribution of classes of drugs administered.

	Percentage
ACEIs/ARBs	66.90%
Spironolactone	50.70%
Oral loop diuretics	92.25%
Beta-blockers	77.46%
Digoxin	22.53%
All treatments	9.15%
ACEIs/ARBs and oral loop diuretics	61.26%

**Table 7:** Logistic regression applied to marked limitation of physical activity based on H2FPEF score.

Dep. Variable:	Marked Limitation of Physical Activity	No. of Observations:	126			
**Model:**	Logit	**Df residuals:**	124			
**Method:**	MLE	**Df model:**	1			
**Date:**	Monday, 7 April 2025	**Pseudo R-squ.:**	0.1069			
**Converged:**	True	**LL-Null:**	−73.471			
	**Coef.**	**Std err.**	**z**	**P > |z|**	**[0.025**	**0.975]**
**Intercept:**	−2.1784	0.876	−2.487	0.013	−3.895	−0.462
**Heart failure with preserved ejection fraction:**	0.5708	0.161	3.543	0	0.255	0.887

**Table 8:** Logistic regression applied to pitting edema based on H2FPEF scores.

Dep. Variable:	Pitting Edema	No. of Observations:	126			
**Model:**	Logit	**Df residuals:**	124			
**Method:**	MLE	**Df model:**	1			
**Date:**	Mon, 7 April 2025	**Pseudo R-squ.:**	0.06136			
**Converged:**	True	**LL-Null:**	−85.037			
	**Coef.**	**Std err.**	**z**	**P > |z|**	**[0.025**	**0.975]**
**Intercept:**	−2.6554	0.762	−3.487	0	−4.148	−1.163
**Heart failure with preserved ejection fraction:**	0.381	0.123	3.108	0.002	0.141	0.621

**Table 9:** Logistic regression applied to fatigue based on H2FPEF scores.

Dep. Variable:	Recent Fatigue	No. of Observations:	126			
**Model:**	Logit	**Df residuals:**	124			
**Method:**	MLE	**Df model:**	1			
**Date:**	Mon, 7 April 2025	**Pseudo R-squ.:**	0.02715			
**Converged:**	True	**LL-Null:**	−74.446			
	**Coef.**	**Std err.**	**z**	**P > |z|**	**[0.025**	**0.975]**
**Intercept:**	−0.5437	0.776	−0.7	0.484	−2.065	0.978
**Heart failure with preserved ejection fraction:**	0.2615	0.134	1.95	0.051	−0.001	0.524

**Table 10:** Logistic regression applied for regular exercise based on H2FPEF score.

**Dep. variable:**	Sport	**No. of observations:**	126			
**Model:**	Logit	**Df residuals:**	124			
**Method:**	MLE	**Df model:**	1			
**Date:**	Mon, 7 April 2025	**Pseudo R-squ.:**	0.04229			
**Converged:**	True	**LL-Null:**	−70.3			
	**Coef.**	**Std err.**	**z**	**P > |z|**	**[0.025**	**0.975]**
**Intercept:**	0.7904	0.82	0.963	0.335	−0.818	2.398
**Heart failure with preserved ejection fraction:**	−0.3371	0.145	−2.323	0.02	−0.621	−0.053

**Table 11:** Logistic regression applied for sadness based on H2FPEF scores.

Dep. Variable:	Sadness	No. of Observations:	126			
**Model:**	Logit	**Df residuals:**	124			
**Method:**	MLE	**Df model:**	1			
**Date:**	Mon, 7 April 2025	**Pseudo R-squ.:**	0.04744			
**Converged:**	True	**LL-Null:**	−78.742			
	**Coef.**	**Std err.**	**z**	**P > |z|**	**[0.025**	**0.975]**
**Intercept:**	−1.2249	0.768	−1.595	0.111	−2.73	0.28
**Heart failure with preserved ejection fraction:**	0.3481	0.134	2.606	0.009	0.086	0.61

**Table 12:** Logistic regression applied for palpitations based on H2FPEF scores.

Dep. Variable:	Palpitations	No. of Observations:	126			
**Model:**	Logit	**Df residuals:**	124			
**Method:**	MLE	**Df model:**	1			
**Date:**	Mon, 7 April 2025	**Pseudo R-squ.:**	0.04795			
**Converged:**	True	**LL-Null:**	−94.79			
	**Coef.**	**Std err.**	**z**	**P > |z|**	**[0.025**	**0.975]**
**Intercept:**	−2.4777	0.72	−3.442	0.001	−3.888	−1.067
**Heart failure with preserved ejection fraction:**	0.3374	0.115	2.929	0.003	0.112	0.563

**Table 13:** Relationship between the H2FPEF score, the quality-of-life factors, and the NT-pro BNP.

Dependent Variable	r-Squared	Independent Variable	Coef.	*p*-Value
Pitting edema	0.06136	Heart failure with preserved ejection fraction	0.381	0.002
Marked limitation of physical activity	0.1069	Heart failure with preserved ejection fraction	0.5708	0
Sadness	0.04744	Heart failure with preserved ejection fraction	0.3481	0.009
Ability to exercise	0.04229	Heart failure with preserved ejection fraction	−0.3371	0.02
Palpitations	0.04795	Heart failure with preserved ejection fraction	0.3374	0.003
Recent fatigue	0.02715	Heart failure with preserved ejection fraction	0.2615	0.051
NT-pro BNP	0.025	Heart failure with preserved ejection fraction	537.8486	0.06

## Data Availability

The data presented in this study are available from the corresponding author upon request. The data are not publicly available due to privacy issues.

## Abbreviations

The following abbreviations were used in this manuscript:

ACEIs	Angiotensin-converting enzyme inhibitors
ARBs	Angiotensin receptor blockers
HF	Heart failure
HFPEF	Heart failure with preserved ejection fraction
KCCQ	The Kansas City Cardiomyopathy Questionnaire
LV	Left ventricular
LVEDVs	Left-ventricular end-diastolic volumes
LVEF	Left-ventricular ejection fraction
LVESVs	Left-ventricular end-systolic volumes
MLHFQ	The Minnesota Living with Heart Failure Questionnaire
NYHA	New York Heart Association
NT-proBNP	N-terminal prohormone of brain natriuretic peptide
